# Evaluation and significance of hyperchromatic crowded groups (HCG) in liquid-based paps

**DOI:** 10.1186/1742-6413-4-2

**Published:** 2007-01-22

**Authors:** Mamatha Chivukula, R Marshall Austin, Vinod B Shidham

**Affiliations:** 1 Department of Pathology, Magee-Womens Hospital 300 Halket Street Pittsburgh, PA 15213-3180 USA; 2 Medical College of Wisconsin, Milwaukee, USA

## Abstract

**Objective:**

Hyperchromatic crowded groups (HCG), a term first introduced into the cytology literature by DeMay in 1995, are commonly observed in Pap tests and may rarely be associated with serious but difficult to interpret lesions. In this study, we specifically defined HCG as dark crowded cell groups with more than 15 cells which can be identified at 10× screening magnification.

**Methods:**

We evaluated consecutive liquid-based (Surepath) Pap tests from 601 women (age 17–74 years, mean age 29.4 yrs) and observed HCG in 477 cases. In all 477 HCG cases, Pap tests were found to be satisfactory and to contain an endocervical sample. HCG were easily detectible at 10× screening magnification (size up to 400 um, mean 239.5 um) and ranged from 1 to 50 (mean 19.5) per Pap slide.

**Results:**

HCG predominantly represented 3-Dimensional groups of endocervical cells with some nuclear overlap (379/477 – 79%), reactive endocervical cells with relatively prominent nucleoli and some nuclear crowding (29/477 – 6%), clusters of inflammatory cells (25/477 – 5.2%), parabasal cells (22/477 – 4.6%), endometrial cells (1/477 – 0.2%). Epithelial cell abnormalities (ECA) were present in only 21 of 477 cases (4.6%). 18 of 21 women with HCG-associated ECA were less than 40 years old; only 3 were =/> 40 years. HCG-associated final abnormal Pap test interpretations were as follows: ASCUS (6/21 – 28%), LSIL (12/21 – 57%), ASC-H (2/21 – 9.5%), and HSIL/CIN2-3 (3/21 – 14%). The association of HCG with ECA was statistically significant (p = 0.0174. chi-square test). In patients with ECA, biopsy results were available in 10 cases, and 4 cases of biopsy-proven CIN2/3 were detected. Among these four cases, HCG in the Pap tests, in retrospect represented the lesional high grade cells in three cases (one HSIL case and two ASC-H cases). Interestingly, none of the 124 cases without HCG were found to have an epithelial cell abnormality.

**Conclusion:**

We conclude: **a. **HCG are observed in a high proportion of cervical smears. **b**. In the vast majority of cases, HCG are benign. **c. **ECA were only observed in cases with HCG. This observation is consistent with the hypothesis that the presence of HCG in Pap tests most often represents adequate sampling of the transformation zone, thus increasing the chances of detecting an epithelial cell abnormality. **d. **Only a few cases with HCG were associated with a serious ECA, but careful scrutiny of all HCG appears warranted to avoid the potential diagnostic pitfall of a significant false negative interpretation.

## Background

The term hyperchromatic crowded groups (HCG) was introduced into the cytology literature in DeMay's 1995 first edition of The Art and Science of Cytopathology [1]. DeMay noted that "although hyperchromatic crowded groups are usually benign and include such entities as endometrial cells and severe atrophy; they can also be the general cytologic appearance of a variety of serious lesions." He first noticed the possible significance of these groups when examining a large series of Pap tests interpreted as "negative" from a single patient who ultimately developed cervical cancer and initiated litigation [2]. Glandular cells tend to present much more frequently as HCG than squamous cells. Endocervical cells, endometrial cells, tubal metaplasia, and cone artifact are just some of the many benign glandular cell groups which may present as HCG, whereas atrophy is the most common benign cause of HCG associated with squamous epithelium [1,3]. Although relatively uncommon, epithelial cell abnormalities (ECA) may also present as HCG. Significant lesions such as carcinoma-in-situ (CIS), squamous cell carcinoma, adenocarcinoma in situ (AIS), endocervical adenocarcinoma, small cell carcinoma, endometrial adenocarcinoma, and even metastatic carcinoma may present in part or entirely as HCG [1,3,4].

There is currently limited literature as how to precisely differentiate benign HCG from precancerous or malignant HCG [1,3,4]. The difficulty of cytologic interpretation may on occasion represent a highly clinically significant diagnostic pitfall. Aside from seminal publications by DeMay, reports specifically analyzing the cytologic evaluation of HCG are still uncommon [5-7]. We undertook this study to further evaluate this challenge and to characterize some of the cytologic patterns associated with HCG.

## Materials and methods

We define HCG in this study as groups of darkly staining cells which measured 100 μm or more and which were easily detectable at 10× screening magnification. Consecutive Pap tests from 601 women from 17 to 74 years old (mean 29) were selected for the study. All specimens were collected with the Cervex-Brush^® ^in SurePath collection medium™. Of the 601 consecutive Pap tests, 38 had no endocervical cells or T zone metaplastic cells and 10 were unsatisfactory. The Pap slides were evaluated for the presence of HCG. Cytomorphologic features such as the size of the cellular groups, background, cohesiveness of the cell clusters, shape of the cell groups, presence of feathering or other cell group border features were noted. The individual size and shape of the cells, N/C ratio, cytoplasm, chromatin, nuclear contour, and presence of nucleoli were also noted.

## Results

**Figure 1:**
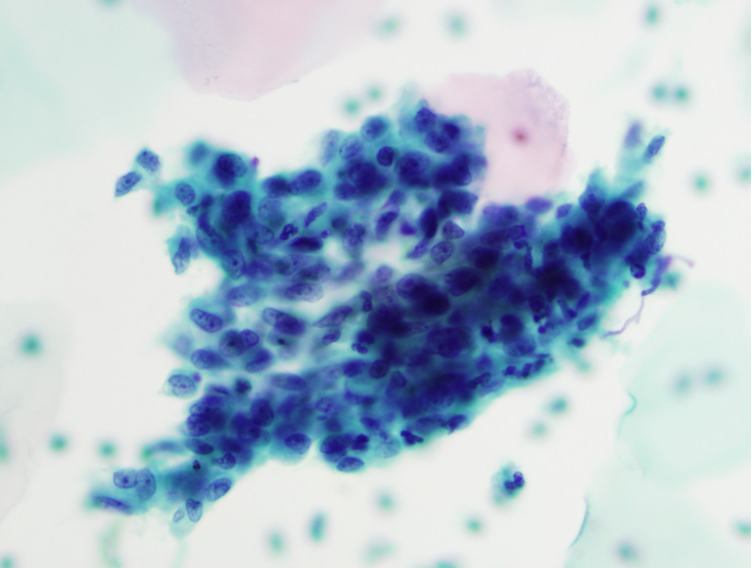
3-Dimensional groups of endocervical cells with some nuclear overlap, (40×).

**Figure 2:**
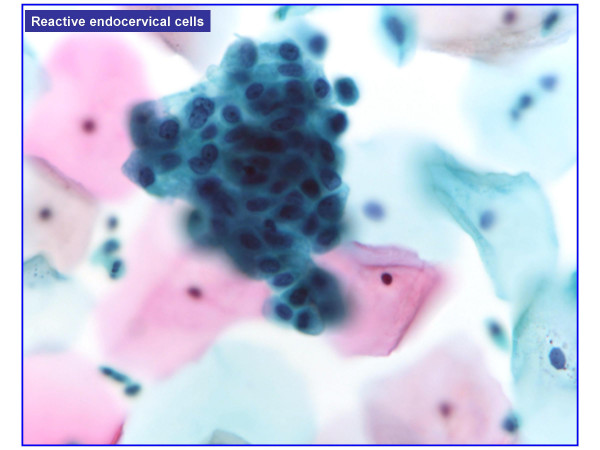
Reactive endocervical cells with relatively prominent nucleoli and some nuclear crowding (100×).

**Figure 3:**
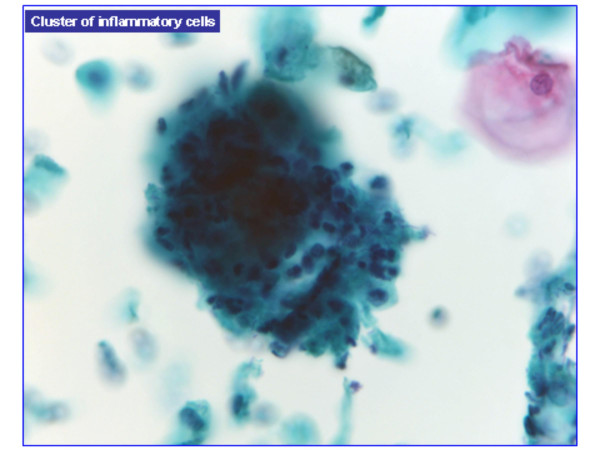
Groups of inflammatory cells, (100×).

**Figure 4:**
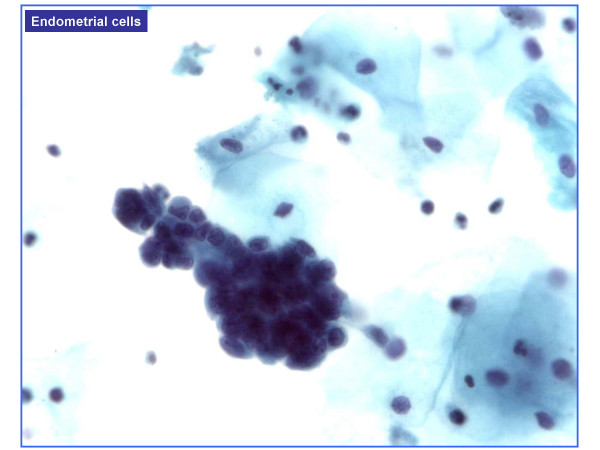
Endometrial cells, (100×).

**Figure 5:**
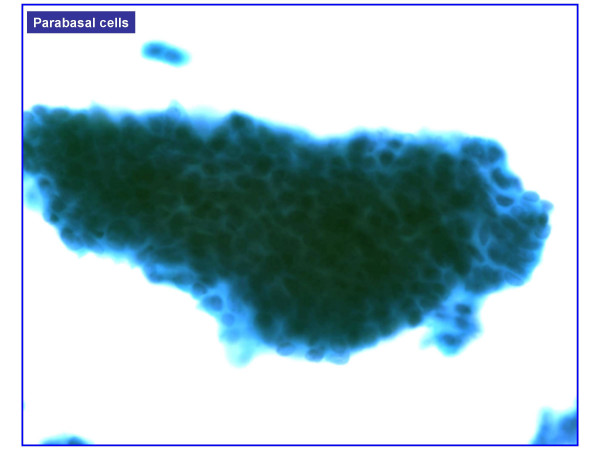
Parabasal cells, (100×).

HCG were present in 477 of 601 cases (**79.4%**) and absent in 124 cases (**20.6%**). HCG were benign in over 95% of cases (Table 1). Benign HCG included 3-Dimensional groups of endocervical cells with some nuclear overlap (379/477 – 79%) (Figure 1), reactive endocervical cells with relatively prominent nucleoli and some nuclear crowding (29/477 – 6%) (Figure 2), groups of inflammatory cells (25/477 – 5.2%) (Figure 3), endometrial cells (1/477 – 0.2%) (Figure 4) and parabasal cells (15 atrophic and 7 postpartum) (22/477 – 4.6%) (Figure 5).

**Table 1:** Association of HCG with benign groups

**Groups identified**	**#/out of**	**%**
**3-Dimensional groups of endocervical cells with some nuclear overlap**	379/477	79.4
**Reactive endocervical cells with relatively prominent nucleoli and some nuclear crowding**	29/477	6.0
Endometrial cells	1/477	0.2
Parabasal cells (15 atrophic and 7 postpartum)	22/477	4.6
Clusters of inflammatory cells	25/477	5.2
***Total***	456/477	95.4

**Figure 6:**
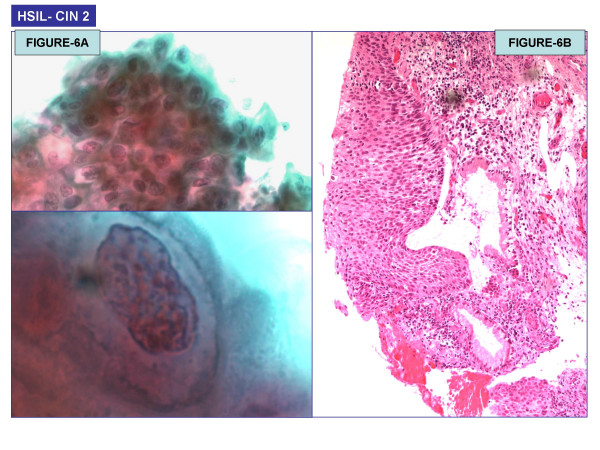
(A): HSIL (Pap Test) (100×); (B): Cervical intraepithelial neoplasia 2 (CIN2) on biopsy (40×).

**Figure 7:**
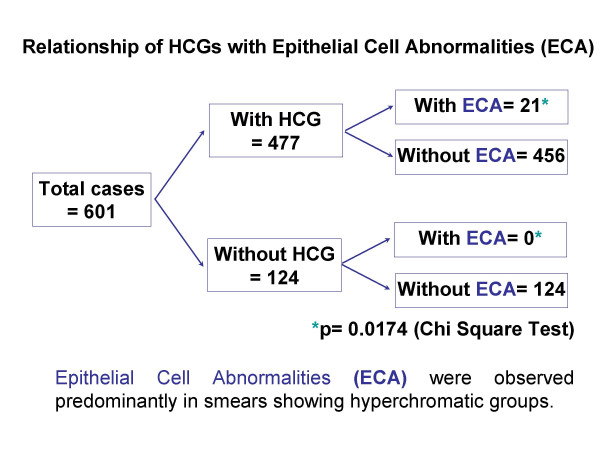
Relationship of HCG with Epithelial abnormalities (ECA).

Twenty one of 477 HCG cases (4.6%) were associated with epithelial cell abnormalities (ECA). 18 of 21 women with HCG-associated ECA were below 40 years of age; only 3 were =/> 40 years (Figure 7). The final abnormal cytological interpretations/results in these abnormal cases were as follows: ASCUS (6/21 – 28%), ASC-H (2/21 – 9.5%), LSIL (12/21 – 57%), and HSIL (3/21 – 14%). The association of HCG with ECA was statistically significant (p = 0.0174, chi-square test). In the group with ECA, follow-up cervical biopsy results were available in 10 cases. One LSIL case with non-lesional benign HCG was found subsequently on follow-up cervical biopsy to harbor a high grade squamous intraepithelial lesion (CIN II) (Table 2). A total of four biopsy proven CIN2/3 cases were associated with HCG (Figure 6). Among these four cases, HCG in the smear actually represented the atypical lesional cells in three cases (one HSIL cytology and two ASC-H cytology cases). Although the total number of HCG-associated abnormal cases in this series is small, an interesting and unexpected finding was that in none of the 124 cases with satisfactory sampling of transformation zone but without HCG showed any epithelial cell abnormality.

**Table 2:** Cyto-histo correlation of HCG representing HSIL

Total # cases	Cytologic interpretation	Biopsy interpretation
1	LSIL	CIN-2
1	HSIL/CIN-2	CIN-2
2	ASC-H	CIN-2

## Discussion

Hyperchromatic crowded groups, termed HCG by DeMay [1], are a frequent occurrence in Pap Tests. Benign glandular cells HCG are seen far more frequently than either abnormal glandular cell HCG or squamous cell HCG, normal or abnormal in routine Pap Tests. In this study, *endocervical cells *were by far the most common cause of benign HCG cell groups. We report here for the first time the significant association of HCG with endocervical sampling. All cases with HCG were consistent with optimum sampling of the transformation zone. We also report for the first time a significant increase in detection of epithelial cell abnormalities (ECA) in Pap tests with HCG as opposed to Pap tests without HCG. We conclude that these observations are best explained by the hypothesis that the presence of HCG in Pap tests most often represents adequate sampling of the transformation zone, thus increasing the chances of detecting an epithelial cell abnormality.

Endocervical sampling has been significantly enhanced with the advent of the endocervical brush [8], even as coincidentally increased sampling of the lower uterine segment (LUS) [9,10], endocervical tubal metaplasia (TME) [11,12], brush-induced atypia (brush effect) [13], reparative endocervical changes [14], cone artifact [15], cervical endometriosis [16], and microglandular hyperplasia [17,18] have added to the complex list of benign endocervical glandular HCG that must be distinguished from neoplastic lesions. The detection of TME, approximately 31% in cone or hysterectomy specimens [19], has undoubtedly risen in recent decades as a result of the improved ability of the cytobrush to sample the upper endocervix. TME may present as flat sheets or 3D HCG with a finely granular chromatin, dark nuclei, absent nucleoli. *The presence of cilia and peg cells are very helpful*. In the absence of ciliated cells, the reporting of atypical glandular cells on the Pap tests is a common outcome [11,12].

*Benign endometrial cells *often present in the Pap tests as HCG. The cells are small, and crowded; the nuclei may be degenerate and hyperchromatic. Mitotic figures are not typically seen in exfoliated cells but may be seen in directly sampled cells [3]. Benign endometrial cell HCG spontaneously shed in menstrual smears may especially show concerning degenerative chromatin changes, but the cell groups typically lack features commonly described in neoplasia [20], especially the more pronounced nuclear atypia seen at least in higher grade endometrial cancers [21]. *Tubal metaplasia of the endometrium and other forms of endometrial metaplasia, also termed epithelial cytoplasmic change *[22], have been much more frequently recognized in endometrial surgical pathology specimens than in cytology specimens. The relatively few cytologic descriptions suggest that they may present as HCG and that their accurate recognition can be a significant diagnostic challenge [23,24].

One of the most important abnormal glandular causes of HCG is endocervical neoplasia, including both *adenocarcinoma in situ *(AIS) and invasive adenocarcinoma [1,3]. Detection rates of AIS on Pap tests have risen with the widespread use of endocervical brush sampling [25]; however, there are many benign conditions which can mimic AIS [26]. Additional features such as marked hyperchromasia, altered nuclear polarity, increased N/C ratio, and associated single cells have been described as helpful diagnostic features [26,27]. An increasingly common endometrioid variant of AIS has a distinct small cell pattern and may be an especially common cause of false negative interpretations as it can be difficult to distinguish from cells sampled from LUS [28]. Presence of endometrial glands and stroma in the cells derived from LUS are helpful cytologic features to distinguish LUS from AIS. Endometrial adenocarcinoma can also present as HCG. Nuclear enlargement and presence of nucleoli are helpful cytomorphologic features (28). Some studies have reported enhanced accurate recognition with liquid-based cytology [30-33].

Parabasal cells associated with an atrophic cellular pattern in postmenopausal or postpartum specimens are often seen as syncytial aggregates of cells with small closely packed parabasal cells with dark nuclei and scant cytoplasm. These may on occasion be difficult to distinguish from CIS [34]. Maturation of the cells at the periphery of the groups of parabasal cells is one clue which may help to recognize the atrophic nature of the smear. A study by *Harris et al *to evaluate the dense nuclei of HCG using digital measurements revealed that the atrophic groups had significantly lower nuclear area (mean 0.4 μm) than HSIL (mean 0.9 μm) [6]. In another recent study on the cytomorphologic spectrum of ASC-H from the authors' aboratories, it was noted that *parabasal cell HCG often present *as small dark nuclei with variable cytoplasm and a moderate N/C ratio. The chromatin is typically dark but not clumped. These groups may be interpreted as ASC-H [35]. Recognition of this distinctive cytomorphologic pattern and HPV DNA testing can be very helpful in establishing the benign character of these cell groups [36].

The other most clinically significant differential diagnosis with HCG is that of a high grade squamous intraepithelial lesion (CIN2,3/CIS) with or without involvement of endocervical glands. The cytomorphologic features of HSIL include small dysplastic cells as compared to larger cells of LSIL. HCG in this study never represented lesional LSIL cells. Cells of HSIL usually occur as small single cells, but when present as syncytial-like aggregates, the HCG may be misinterpreted as other benign HCG. Close scrutiny under high-magnification is needed. Attention must be given to features such as increased nuclear size and hyperchromasia, fine-coarsely granular chromatin, and irregular nuclear contours. Nucleoli are usually absent but may be seen along the periphery of the groups, especially when the dysplasia involves endocervical glands [37]. In Liquid based preparations, it has been observed that dispersed single dysplastic cells are more often seen than sheets or syncytial groups [38]. The study of dense HCG by *Harris et al *found that HSIL groups contained the highest nuclear area (mean-0.9 μm) as compared to invasive squamous cell carcinoma (mean -0.8 μm) or endometrial adenocarcinoma (mean – 0.64 μm) [6]. Interpretation of HCG of HSIL may be challenging in conventional as well as liquid based preparations. In a recent study from the College of American Pathologists PAP Program, poorly performing conventional smear and Thin-Prep HSIL cases were compared. HCG were observed in both the preparations. The HSIL groups were often interpreted as glandular lesions when there was rounding up of cells with small nuclei and smooth contoured borders. On the other hand, these cell groups were interpreted as squamous lesions when elongated and large nuclei were present [38,39]. The distinction of CIS involving the endocervical glands from AIS may impact management decisions. HCG may be seen in both the groups. The cells of AIS show spindling in the center with round to oval nuclei, peripheral palisading, and pseudostratification at the edge [40].

In summary, HCG are observed in a high proportion in cervical smears. Most of these cell groups turn out to be benign. However, there is a small proportion of HCG associated with serious abnormalities. The careful scrutiny of HCG is mandatory, as such cell groups are common in litigated cases alleged as interpretive false negatives [3]. A number of different cytomorphologic features, discussed above, can be utilized in distinguishing benign from high grade precancerous or malignant HCG. The judicious use of reflex HPV-DNA testing (34), p16^INK4A ^or other immunocytochemical stains such as ProEx C [42], and computer-assisted imaging employing DNA sensitive stains [43] may aid interpretation of HCG-associated high grade squamous and glandular intraepithelial lesions or malignancy.

## Abbreviations

AIS – adenocarcinoma in-situ; CIS-carcinoma-in situ; DNA, Deoxyribose nucleic acid; HCG – Hyperchromatic crowded groups; HPV, Human papilloma virus; LUS, Lower uterine segment; TME, Tubal Metaplasia of Endocervix;

## Competing interests

The author(s) declare that they have no competing interests.

## Authors' contributions

(MC): Faculty collected all the data, participated in cytological evaluation, and drafting of manuscript, presentation of the data at EUROGIN conference.

(RMA): Faculty, drafting of manuscript, manuscript writing.

(VS) Mentor, conceptual organization, cytological-histological evaluation, and manuscript writing.
